# Cold Knife Hysteroscopic Myomectomy: A Literature Review on Its Role as a Fertility Sparing Tool

**DOI:** 10.3390/healthcare13141651

**Published:** 2025-07-09

**Authors:** Giuseppe Gullo, Valentina Billone, Erika Catania, Giulia Russo, Stamatios Petousis, Lina De Paola, Elena Chitoran, Gaspare Cucinella, Simone Ferrero

**Affiliations:** 1 Department of Obstetrics and Gynaecology, AOOR Villa Sofia–Cervello, University of Palermo, 90133 Palermo, Italy; valentina.billone@gmail.com (V.B.); erika.catania0702@gmail.com (E.C.); giuliarusso779@gmail.com (G.R.); gasparecucinella1@gmail.com (G.C.); 2 2nd Department of Obstetrics and Gynaecology, Aristotle University of Thessaloniki, 541 24 Thessaloniki, Greece; petousisstamatios@gmail.com; 3 Department of Anatomical, Histological, Forensic and Orthopedic Sciences, Sapienza University of Rome, 00161 Rome, Italy; lina.depaola@uniroma1.it; 4 General Surgery and Surgical Oncology Department I, Bucharest Institute of Oncology “Al. Trestioreanu”, 022328 Bucharest, Romania; chitoran.elena@gmail.com; 5 Department of Neurosciences, Rehabilitation, Ophthalmology, Genetics, Maternal and Child Health (DiNOGMI), University of Genoa, 16132 Genoa, Italy; simone.ferrero@unige.it; 6 Academic Unit of Obstetrics and Gynecology, IRCCS Ospedale Policlinico San Martino, 16132 Genoa, Italy

**Keywords:** cold knife hysteroscopic myomectomy, resectoscopy, fertility sparing, fibroids

## Abstract

Cold knife hysteroscopic myomectomy (CKHM) is a minimally invasive surgical technique used to remove submucosal fibroids, commonly associated with infertility. Methods: A comprehensive literature search was conducted across databases including PubMed, Scopus, and Google Scholar, focusing on studies published from 2011 to 2024. The aim of this narrative review was to highlight the minimally invasive cold loop resectoscopic approach for the treatment of submucosal fibroids. Results: The review revealed that CKHM is associated with favorable fertility outcomes, with studies reporting increased pregnancy rates; most studies indicated that CKHM did not negatively affect endometrial function. Conclusions: Cold knife hysteroscopic myomectomy is a safe and effective option for women with submucosal fibroids who wish to preserve fertility due its minimal invasiveness and low complication rates.

## 1. Introduction

Fibroids, also known as leiomyomas or myomas, are the most common condition affecting the female reproductive system. They are benign tumors that arise from the proliferation of smooth muscle cells in the myometrium [1,2]. Uterine fibroids arise from the benign monoclonal division of myometrial cells. Their extracellular matrix includes collagen, fibronectin, and proteoglycans. Myomas can cause a wide range of gynecological and obstetric symptoms, including abnormal uterine bleeding, pelvic pain, infertility, placental abruption, preterm birth, and postpartum hemorrhage [3,4,5]. In 50% of cases, they are asymptomatic [6]. The prevalence of this condition is difficult to estimate as it varies based on age, ethnicity, symptoms, and diagnosis. Determining the incidence and prevalence of uterine fibroids is difficult. Some studies [1,2] indicate a prevalence of up to 70–80% in women aged 50, but this varies based on factors such as age, family history, and ethnicity. Among Scandinavian women aged 33 to 40, the prevalence is 7.8%, while in the United States, it is nearly 40% in white women and over 60% in women of African descent within the same age group. The group most affected by uterine fibroids is African American women, with an incidence two to three times higher than in Caucasians. It is estimated that more than 50% of women over the age of 45 have this condition [7].

While the exact mechanism behind the formation of fibroids remains unclear, the pathogenesis appears to be influenced by the interaction between ovarian hormones, genetic factors, and various other risk elements. Elevated levels of estrogen and progesterone stimulate mitotic activity, leading to cellular proliferation. Another hypothesis suggests that fibroids may also result from ischemic tissue damage, which releases growth factors that trigger cell division and promote fibroid development. Basic fibroblast growth factor (bFGF), epidermal growth factor (EGF), and transforming growth factor beta 3 (TGF-β3) are some of these growth factors [3,8].

Various treatment strategies are available, depending on both the characteristics of the fibroid and the patient-specific factors. These include pharmacological treatments such as progestins, danazol, GnRH analogs [4], intrauterine devices, and progesterone receptor modulators, as well as procedural approaches like hysteroscopic, resectoscopic, and surgical techniques, including both laparoscopic and laparotomic approaches.

Numerous studies [9,10,11,12,13] have investigated the effects of cold knife myomectomy on female fertility, generally with favorable outcomes. Removing fibroids can improve fertility in women suffering from infertility related to fibroids, reducing the risk of complications during pregnancy and increasing the chances of conception. However, the results of cold knife myomectomy depend on various factors, including the size, number, and location of the fibroids. The recurrence of fibroids can also impact long-term fertility. However, it is essential that each case be evaluated individually, taking into account the specific characteristics of the fibroids and the patient’s overall health and fertility.

In particular, this narrative review will focus on the minimally invasive cold loop resectoscopic approach for the treatment of submucosal fibroids.

## 2. Materials and Methods

A literature search was conducted on PubMed and Google Scholar using the terms ‘COLD KNIFE MYOMECTOMY’, ‘COLD KNIFE RESECTOSCOPY’, ‘COLD LOOP MYOMECTOMY’, and ‘COLD LOOP HYSTEROSCOPIC MYOMECTOMY’. The selected articles spanned a period of 13 years, from 2011 to 2024.

Only papers written in English were included. Initially, we reviewed the titles and abstracts if the study appeared potentially relevant. We found data from case reports, randomized trials, and systematic reviews that were pertinent to the research topic, involved human data, and consisted of either reviews or studies with empirical data.

This review aimed to analyze the benefits of a minimally invasive cold loop resectoscopic approach for the treatment of submucosal fibroids, while also identifying potential gaps in the existing research.

## 3. Results

The studies we analyzed focused on the applications, effectiveness, and outcomes of cold knife myomectomy. As described by Leone et al. [14], this method could be a safe and viable option for treating intramural/submucosal myomas, particularly in patients aiming to preserve fertility. Furthermore, the use of non-electrical tools minimizes damage to surrounding tissues and enhances the precision of the procedure, with a low complication rate and quick recovery post-surgery [14,15]. In particular, one article described the superiority of cold loop myomectomy compared to other approaches in terms of the risk of post-procedure intrauterine adhesions [14].

Mazzon et al. [15] aimed to estimate the prevalence and characteristics of intrauterine adhesions following cold loop resectoscopic myomectomy in a retrospective study involving 688 women, each with one or more G1–G2 myomas. All patients underwent cold loop resectoscopic myomectomy, followed by diagnostic hysteroscopy two months after surgery. A total of 806 myomas were removed, with each surgery involving the removal of one to five fibroids. Complications occurred in eight cases (1.16%). No hemorrhages, intravasation syndrome, or perforation were recorded with the use of the cold loop. Intrauterine adhesions were observed in twenty-nine patients (4.23%); two of them required additional hysteroscopic surgery to remove fibrous adhesions, while twenty-seven patients had mild adhesions removed in outpatient hysteroscopy. No intrauterine devices or anti-adhesion treatments were used at the end of the surgery.

Another article [16] aimed to evaluate the safety and effectiveness of cold loop hysteroscopic myomectomy in a large cohort of patients. This study, published by Mazzon and Favilli et al. [16], involved 1215 patients with one or more G1-G2 submucosal myomas treated with cold loop hysteroscopic myomectomy. A total of 1690 myomas were removed, with each surgery involving the removal of one to five fibroids. Of the 1215 patients, 1017 (83.7%) were treated with a single surgery. Twelve intraoperative complications occurred (0.84%). There were no reported cases of uterine perforation or clinical intravasation syndrome during the procedure. The use of the cold loop technique in resectoscopic myomectomy was associated with a low rate of minor complications and no major complications, making it especially relevant for fertility preservation and future pregnancies.

In this regard, Mazzon also described a case report involving a 28-year-old infertile woman [17] diagnosed with diffuse uterine fibromatosis and episodes of metrorrhagia. Ultrasound revealed numerous subserosal, intramural, and submucosal myomas. Diagnostic hysteroscopy showed a uterine cavity entirely distorted by myomas. A two-step “cold loop” hysteroscopic myomectomy was performed following the previously described technique. One month post-treatment, no submucosal myomas remained, and the uterine cavity appeared normal and free of adhesions on endoscopy. After the procedure, the patient successfully carried three consecutive uncomplicated pregnancies to term. The authors suggested that cold loop resectoscopic myomectomy offers new promising possibilities for women with diffuse uterine leiomyomatosis who wish to conceive.

Another case report by Mazzon, Favilli, and Grasso et al. described a 28-year-old woman with a 9-month history of heavy periods and anemia [18]. A transvaginal ultrasound revealed a single 56-mm myoma with a 1.7-mm myometrial margin. Hysteroscopy revealed a 40-mm G1 myoma in the uterine cavity. After counseling, she opted for hysteroscopic myomectomy, preceded by treatment with gonadotropin-releasing hormone analog (GnRHa), which reduced the fibroid size to 34 mm. During surgery, the myoma was not visible initially but emerged when the intrauterine pressure was reduced. A one-step cold loop resectoscopic myomectomy was performed. The intracavitary component of the myoma was removed using conventional slicing techniques until the cleavage plane was reached. A non-electrified metallic loop (cold loop) was then used to bluntly dissect and remove the myoma without damaging the surrounding myometrium. The myoma was successfully extracted in its entirety with no complications.

The patient was discharged the following day. A follow-up hysteroscopy performed two months later showed a normal uterine cavity, free from synechiae or residual myoma tissue. The patient’s menstrual bleeding became regular thereafter. This study suggested that cold loop hysteroscopic myomectomy is a safe and effective method for removing sinking myomas, eliminating the need for ultrasound and minimizing potential risks. Further research with a larger number of patients is required to assess the full benefits of this technique and the role of GnRHa therapy.

In another publication, Mazzon et al. [19] reviewed data from patients treated with cold loop hysteroscopic myomectomy over a seven-year period, assessing the size, number, and grading of myomas and their impact on the procedure’s success. The results showed that the size and number of myomas, along with the patient’s age, were key factors influencing whether the procedure could be completed in one step. Larger and multiple myomas were more likely to require multiple surgeries, while myomas with more than 50% intramural extension (G2) did not significantly affect the procedure’s completion. The study also found that pre-surgical GnRH agonist therapy (used to shrink the fibroids) did not influence the success of the single-step procedure.

The study concluded that cold loop hysteroscopic myomectomy is a safe and effective treatment, even for patients with multiple myomas. While the size and number of fibroids, along with the patient’s age, can affect the procedure’s success, the cold loop technique is a reliable option for minimizing complications and avoiding the need for repeated surgeries. The procedure’s success was also largely dependent on the skill and experience of the surgeon.

As previously mentioned, in 95% of cases, fibroids are located in the uterine body. They can be intramural, meaning within the myometrium, they are submucosal, when they grow beneath the endometrium and deform the cavity, or subserosal, when they develop beneath the uterine serosa [5,20]. Macroscopically, they appear as a collection of elastic fibers with a whorled appearance and a whitish color. Their consistency is variable, and they may sometimes undergo degeneration. Fibroids have a rich vascular network and a pseudocapsule, which is highly vascularized and represents an excellent plane of cleavage during myomectomy. The pseudocapsule results from ischemic phenomena affecting the surrounding structures that the fibroid compresses [5,16,20]. It is important to note that as the fibroid grows, it compresses the myometrial fibers and connective tissue, displacing them but not invading them. Ultrasonographically, this pseudocapsule is recognized by its characteristic hyperechoic ring.

Before undergoing hysteroscopic surgery, it is essential to perform a pelvic ultrasound and a diagnostic hysteroscopy. These evaluations help assess the number, size, grade, and location of fibroids within the uterine cavity, as well as the thickness of the myometrial free margin, and the presence of any other associated intracavitary pathologies. The myometrial free margin refers to the minimum thickness between the outer margin of the fibroid and the uterine serosa. Mazzon et al. [16] suggested that a 10 mm myometrial free margin is sufficient for a safe procedure when performed using the cold loop technique [21].

Diagnostic hysteroscopy should be performed between the seventh and twelfth day of the cycle in women of reproductive age, and at any time in menopausal women. It allows us to evaluate the number, size, location, grading, or percentage of intracavitary development, and consistency of the fibroids.

In this regard, we refer to the Wamsteker classification [22] of submucosal fibroids, which divides them into the following stages:

- **G0**: A completely intracavitary fibroid or pedunculated fibroid, with no intramural extension.
- **G1**: Submucosal fibroid with less than 50% intramural extension.
- **G2**: Submucosal fibroid with 50% or greater intramural extension.

In 2005, Lasmar et al. [23] proposed a new classification for submucosal fibroids: the STEPW classification (Size, Topography, Extension, Penetration, Wall). Each parameter is given a score from 0 to 2 for preoperative resectability (Table 1):

- **Score 0–4 (Group I)**: Low-complexity hysteroscopic myomectomy.
- **Score 5–6 (Group II)**: Complex hysteroscopic myomectomy, with consideration for preoperative GnRH analog therapy or a two-stage surgical procedure.
- **Score 7–9 (Group III)**: Fibroids that should be treated with a non-hysteroscopic technique.

### 3.1. Cold Loop Myomectomy: The Technique

The slicing myomectomy technique is well-known and involves fully removing the fibroid using an electrified loop that gradually “cuts” through the growth. However, this technique often does not allow for a clear distinction between the fibroid and healthy myometrial tissue. This can lead to damage to the myometrial bundles, resulting in increased absorption of the distension fluid, reduced myometrial contractility, and a higher risk of bleeding, with the potential to interrupt the procedure.

The cold loop myomectomy technique was developed by Mazzon in 1995 [24] to avoid damage to healthy myometrial tissue. Cold loop myomectomy takes advantage of the fibroid’s pseudocapsule and the contractile ability and elastic recoil of the myometrial muscle fibers. Using the cold loop, it is possible to mechanically treat the intramural part of the fibroid without the use of electrical energy. The elastic recoil of the undamaged myometrial fibers helps remove the intramural portion of the fibroid and facilitates hemostasis without the need for coagulative electricity [16].

Regarding instrumentation, it is recommended to use a 0° optical system and a 26 Fr hysteroscope. For the initial slicing phase, several authors have reported good surgical outcomes using a bipolar current and saline solution [25]. For enucleating the intramural portion, non-electrified cold loops are used, which, due to their sturdiness, apply the mechanical stress necessary to complete the myomectomy. There are different types of cold loops: flat, rake-shaped, and hook-shaped.

To treat submucosal fibroids with an intramural component [15,17,24], the following hysteroscopic steps should be followed:

- Resection of the intracavitary component of the fibroid: This step is performed using the classic slicing technique. The cutting current is applied to reach the endometrial plane while avoiding damage to it. Fibroid fragments should be gradually removed from the cavity. Slicing allows for the opening of the pseudocapsule and facilitates the sliding of the fibroid towards the uterine cavity.
- Enucleation of the intramural component with a cold loop: At this point, the mechanical force of the cold loop is applied to the cleavage plane between the fibroid and healthy myometrium. Enucleation is facilitated by the contraction of the myometrial fibers, which help separate the fibroid from the surrounding tissue.
- Resection of the previously enucleated intramural component: Once the intramural portion of the fibroid has been pushed into the cavity, the slicing technique with a cutting current is applied again. This process is continued until complete enucleation is achieved.
- Checking the integrity of the uterine cavity: Finally, the integrity of the uterine cavity is assessed to ensure that no tissue remnants or damage remain.

### 3.2. Risk of Complications

Among resectoscopic surgeries, myomectomy has the highest incidence rate, with a complication rate ranging from 0.8% to 2.6% [26]. The cold loop technique better respects the anatomical planes and preserves the functional integrity of the myometrium. Unlike the electrified loop technique, complications such as uterine perforation or intravasation syndrome are not typically reported with the cold loop technique. In a large series of 1434 resectoscopic myomectomies performed using the cold loop technique—currently the largest reported by a single center in the literature—the complication rate was 1.47% (21/1434 cases), with only 0.84% of complications being related to the surgical procedure itself.

Hemorrhage is a known complication following resectoscopic myomectomy with the electrified loop [27]. However, the cold loop technique ensures adequate hemostasis and avoids damage to endometrial vessels during capsule enucleation.

Although concerns about bleeding risks have led many gynecologists to hesitate in adopting cold knife hysteroscopic myomectomy (CKHM), experienced hysteroscopists recognize that extensive coagulation is often unnecessary. In fact, the natural contractility of the myometrium, combined with the skillful modulation of intrauterine distension pressure, plays a crucial role in minimizing bleeding and facilitating fibroid removal. These factors highlight the importance of surgical expertise in ensuring both the safety and effectiveness of the procedure. Uterine perforation is the most frequent complication in resectoscopic myomectomy, often occurring during dilation with a Hegar dilator [28,29]. Another stage where perforation can occur is during treatment of the intramural component of the fibroid. The cold loop technique has demonstrated a higher safety rate compared to the electrified loop, which, in the case of perforation, may require laparoscopy to assess the involvement of abdominal vessels or organs [27]. Additionally, there have been reports of spontaneous uterine rupture following perforation with the electrified loop [29,30,31]. In contrast, any perforation caused by the cold loop technique results only in the separation of muscle bundles, with no injury to surrounding organs or bleeding. In such cases, antibiotic therapy and the monitoring of blood parameters are recommended.

Another potential complication of perforation is the spillage of distension fluid into the peritoneal cavity. If this occurs, it is advisable to perform an ultrasound examination at the end of the procedure. If the total fluid volume is less than 1000 mL, draining the hypotonic solution from the peritoneal cavity is recommended.

Intravasation syndrome is another significant complication and can be potentially lethal [32]. For patients undergoing multiple resectoscopic myomectomies, longer surgical times and, consequently, more distention medium being used must be considered. The use of saline (isotonic) combined with bipolar energy is considered safer than the combination of monopolar energy and hypotonic solution because it reduces the risk of hyponatremia [25,33,34]. However, the excessive intravasation of isotonic solution can lead to hypervolemia and fluid overload, resulting in pulmonary edema, hypertension, electrolyte disturbances, arrhythmias, and heart failure [32,35]. The cold loop technique, regardless of the distension medium used, is associated with a lower risk of intravasation syndrome due to fewer injuries to myometrial vessels [36].

Overall, the cold loop technique emerges as a safer alternative to traditional electrosurgical methods, significantly reducing the risk of major intraoperative and postoperative complications, and offering a more conservative and tissue-sparing approach in resectoscopic myomectomy.

### 3.3. Surgical Outcomes

Cold loop myomectomy offers several advantages, including surgical safety, the ability to complete the procedure in a single operative session, and favorable long-term outcomes.

One of the primary concerns in resectoscopic myomectomy is the risk of intravasation of the distension medium, which increases with prolonged surgical times. The longer the surgery, the greater the contact time between the distension medium and injured vessels, leading to an elevated risk of intravasation syndrome. However, the cold loop technique reduces the risk of vessel injury, lowering the risk of intravasation and allowing for longer surgical times if necessary. This can result in the ability to complete the procedure in one operative session.

In a study involving 1244 resectoscopic myomectomies using the cold loop technique for single myomas, the reported rate of completing the procedure in one session was 88.59% for G1 myomas and 82.55% for G2 myomas. Notably, G2 myomas larger than 3 cm were associated with a higher likelihood of requiring multiple procedures [37]. This high success rate demonstrated that the cold loop technique, which respects the uterine anatomical structures, facilitates the complete and effective removal of myomas, ensuring positive surgical outcomes.

As shown by Leone and colleagues, the cold loop technique appears to reduce the significance of the intramural component of submucosal fibroids. In their study, the most complex stage of the procedure was related to myoma size rather than its grade (G1 vs. G2) [14,18]. The intact myometrial structure and preservation of the fibroid’s pseudocapsule contributed to effective uterine contraction, minimizing distension medium absorption and reducing bleeding. These factors make it possible to extend the procedure duration when necessary, without compromising visibility or the ability to complete the surgery in one session.

One common complication following resectoscopic myomectomy is the formation of uterine synechiae (adhesions) [38]. These adhesions can be difficult to treat, especially in women who wish to conceive, as they may interfere with future pregnancies. The primary cause of synechiae is often electrical damage to healthy myometrial tissue, which is more common with the electrified loop technique.

In contrast, the cold loop technique has been associated with a lower rate of uterine adhesions. In a retrospective study of 688 patients undergoing resectoscopic myomectomy with the cold loop technique, postoperative intrauterine synechiae were observed in only 29 patients (4.23%) during hysteroscopic follow-up two months after surgery. Of these, only two patients (0.29%) required surgical intervention for the synechiae, with the remaining patients having their adhesions removed during outpatient hysteroscopy [15]. Notably, one of the two patients with severe adhesions had previously undergone embolization of the uterine fibroids, and the presence of fibrous synechiae was already noted during preoperative diagnostic hysteroscopy.

The cold loop myomectomy technique offers several advantages over traditional methods, including lower complication rates, greater surgical safety, and a higher likelihood of completing the procedure in a single session. It also reduces the risk of uterine synechiae and other adverse outcomes, making it an effective option for women seeking to preserve fertility.

These results confirm that cold loop myomectomy allows for the safe and effective removal of submucosal fibroids—particularly G1 and small-to-medium-sized G2 myomas—in a single operative session in most cases. Moreover, by minimizing trauma to the surrounding myometrial tissue and preserving the pseudocapsule, the technique significantly reduces the risk of intrauterine adhesions and other complications, making it especially suitable for women seeking to maintain or restore fertility.

### 3.4. Obstetrical Complications

Uterine fibroids are a common condition, especially in women of childbearing age. The exact impact of fibroids on fertility, particularly in patients undergoing assisted reproductive technology, remains unclear [9]. While the mechanisms by which fibroids may impair fertility are uncertain and likely multifactorial, current evidence suggests that fibroids, particularly submucosal and larger intramural fibroids, are associated with infertility and poor obstetrical outcomes [10,11,12].

The mechanisms by which fibroids affect fertility include alterations in uterine anatomy, myometrial and endometrial function, as well as changes in endocrine and paracrine molecular signaling. Increased uterine contractility, induced by the presence of fibroids, may also interfere with embryo implantation [11,13,39,40]. However, there are currently no established guidelines for the management of fibroids in infertile women due to the lack of clear scientific evidence on the subject [41,42,43,44].

Despite this, the hysteroscopic removal of submucosal fibroids appears to significantly improve the chances of conception in women with otherwise unexplained infertility [45,46]. It should be noted that there are reports of abnormal placentation in patients who have previously undergone hysteroscopic myomectomy, suggesting that these patients may be at an increased risk of placenta accreta [45,47,48]. The potential underlying mechanism for these complications may be myometrial slicing, which could affect uterine wall integrity and affect placental implantation.

The cold loop technique, which is designed to carefully preserve the anatomical and functional integrity of the endometrium and myometrium, significantly reduces the risks of uterine perforation and the development of post-surgical intrauterine synechiae compared to the traditional electrosurgical loop method [15,16,17,26]. Furthermore, while there are no published studies specifically addressing placentation abnormalities following resectoscopic myomectomy with the cold loop technique, the procedure is expected to carry a lower risk of such complications due to its precision and reduced impact on the surrounding tissue.

Therefore, while further studies are needed to confirm long-term outcomes, the cold loop technique represents a promising and fertility-friendly approach for the surgical management of submucosal fibroids in women desiring pregnancy.

### 3.5. Fertility Outcomes

The presence of myomas in the endometrial cavity is associated with reduced fertility [49], with submucosal fibroids being particularly implicated. It is estimated that approximately 2–12% of infertile women have submucosal uterine fibroids [50]. These types of fibroids are most associated with recurrent miscarriages and implantation failure [51]. The potential mechanisms behind this include distortion of the uterine cavity and altered myometrial function, both of which can negatively impact embryo implantation [52].

Submucosal fibroids are the most frequent fibroids associated with fertility issues, while subserosal fibroids rarely cause such problems [53]. There are limited data on the impact of intramural fibroids on fertility. Several retrospective cohort studies focused on assisted reproduction have shown that women who undergo hysteroscopic myomectomy for fibroid removal have higher clinical pregnancy rates [54].

According to the Practice Committee of the American Society for Reproductive Medicine (2017) and the updated French guidelines [55], myomectomy may be considered in asymptomatic women with cavity-distorting myomas (intramural with a submucosal component or submucosal fibroids) who desire pregnancy, as it may improve pregnancy rates and reproductive outcomes [56,57]. Resectoscopic myomectomy is currently considered the gold standard for treating submucosal fibroids.

Recent studies have shown that preserving the fibroid capsule can improve fertility outcomes, and the cold loop technique appears to help maintain the integrity of the fibroid capsule during myomectomy [38], which may contribute to better fertility outcomes.

Synechiae, or intrauterine adhesions, are the most common postoperative complications following resectoscopic myomectomy [38,58]. They result from trauma to the healthy tissue surrounding the lesion. Intrauterine adhesions are associated with poor reproductive outcomes due to potential tubal and cervical canal obstruction, as well as a reduced endometrial surface area [59]. Furthermore, adhesions are often linked to recurrent pregnancy loss [60].

Several studies have compared the effects of various energy sources used in myomectomy and their impact on adhesion formation [12,17,38]. One study reported a 7.5% rate of intrauterine synechiae in 53 infertile patients after resectoscopic myomectomy performed with bipolar energy, which was associated with fewer risks compared to monopolar energy. The authors attributed the lower rate of synechiae to the use of bipolar energy, which is considered safer and less likely to cause accidental electric fluxes that could damage the healthy myometrium. The cold loop technique, by avoiding thermal damage from electrical energy, further reduces the risk of synechiae formation [61].

Therefore, submucosal fibroids represent a significant factor in female infertility, and their appropriate management is crucial for improving reproductive outcomes. Among the available surgical options, cold loop hysteroscopic myomectomy appears to offer a safe and fertility-preserving alternative, minimizing endometrial trauma and reducing postoperative complications such as intrauterine adhesions.

## 4. Discussion

The surgical treatment of uterine fibroids, particularly submucosal and intramural forms, represents a significant challenge for gynecologists, especially in women who wish to preserve fertility. In this context, hysteroscopic myomectomy using the “cold loop” technique has emerged as a promising and safe approach, demonstrating several advantages over traditional methods based on the use of electrical energy [17,21].

One of the main strengths of the cold loop technique is its safety [16]. Unlike electrical loop myomectomy, which can cause thermal damage to healthy tissue and harm surrounding structures, the cold loop technique minimizes the risk of uterine perforation and intravasation syndrome. These risks are commonly associated with the use of electrical current, which can result in the fusion of surrounding tissues, leading to increased complications such as perforations and blood vessel injuries. In contrast, the cold loop technique better respects the anatomical layers of the myometrium, avoiding damage to blood vessels and muscle fibers, thereby promoting optimal hemostasis without the need for coagulating energy [62].

Preserving the integrity of the myometrium, as achieved with the cold loop technique, is particularly relevant in women seeking fertility preservation [13,59]. By maintaining the integrity of the myometrium and the fibroid pseudocapsule, the procedure allows for better uterine contraction and reduces the risk of post-operative uterine synechiae formation. In fact, several studies have highlighted that the incidence of uterine synechiae following hysteroscopic myomectomy using the cold loop technique is significantly lower compared to traditional approaches utilizing bipolar or monopolar energy. The low incidence of uterine synechiae is crucial, as post-operative adhesions can compromise reproductive capacity, hinder embryo implantation, and even lead to recurrent pregnancy loss [15].

An additional advantage of the cold loop technique is the ability to allow for complete fibroid resection in a single surgical session. This is particularly valuable in patients with moderate-sized fibroids, where other techniques may require multiple procedures or, in some cases, even the use of additional surgical approaches, such as laparoscopy, in the event of complications like uterine perforation [29]. Completing the resection in a single step reduces the risk of complications associated with multiple procedures and enhances treatment efficiency, with a lower psychological and economic impact on the patient.

The evidence gathered so far indicates that the cold loop technique is associated with a very high success rate, particularly for G1 and G2 fibroids [22,23], which are the most common types treated with this technique. However, it is important to note that larger fibroids or those with extensive intramural involvement may present additional challenges, increasing the need for multiple resections. In these cases, the cold loop technique may still represent a beneficial option, minimizing the risk of complications and improving the likelihood of completing the procedure in a single stage [21].

Women with submucosal or intramural fibroids with a submucosal component, who are the most susceptible to fertility issues, can greatly benefit from the resection of these fibroids via hysteroscopic myomectomy [50]. Retrospective studies and clinical trials have shown that the resection of submucosal uterine fibroids leads to significantly higher pregnancy rates in women with unexplained infertility or difficulty achieving pregnancy. This is particularly true when the resection is performed using techniques that preserve the integrity of the uterine cavity and the functionality of the myometrium, such as the cold loop technique.

However, although hysteroscopic myomectomy using the cold loop technique demonstrates an excellent safety profile and favorable long-term outcomes [10], it is crucial to continue gathering data through large-scale studies to better understand the impact of this technique on long-term fertility and obstetric outcomes. Ongoing assessment of the post-operative complications, such as the formation of synechiae and placental abnormalities, remains a key area for research. Nonetheless, current evidence suggests that the cold loop technique represents a preferable choice for women seeking fertility preservation, reducing the risk of significant complications and improving the chances of conception [63,64,65].

In addition to the clinical and surgical aspects, it is important to consider the medico-legal implications associated with the use of cold loop myomectomy [66,67,68]. As with all surgical procedures, it is crucial that a healthcare provider thoroughly and transparently discusses the potential risks and benefits of the procedure with the patient. Obtaining clear and appropriate informed consent is essential, with particular attention given to issues related to future reproductive goals [69,70,71,72,73]. Professional responsibility implies that the physician is aware of the potential risks, including complications such as uterine perforation or synechia formation, and provides clear information about available therapeutic alternatives. A detailed informed consent helps protect the patient’s rights and prevents potential legal disputes arising from misunderstandings regarding the risks of the procedure [74,75,76,77,78]. Furthermore, in the event of complications, it is crucial that medical documentation is accurate and complete to protect both the patient and the professional [79,80,81], ensuring that all stages of decision-making and intervention are managed appropriately and according to the most updated clinical guidelines [82,83,84].

In conclusion, cold loop myomectomy has been proven to be a safe, effective, and well-tolerated technique for the treatment of uterine fibroids, with a positive impact on patients’ fertility outcomes. With further data accumulation and the continued evolution of surgical techniques, this approach is expected to become increasingly standardized in the management of uterine fibroids in women of reproductive ages, improving reproductive outcomes and minimizing surgical risks. However, it is essential that gynecologists and other healthcare professionals remain aware of the medico-legal aspects associated with surgical practice and strive to ensure transparent communication with patients, especially regarding the risks and benefits of the treatment [85].

## 5. Conclusions

In conclusion, cold loop myomectomy offers a promising approach for treating uterine fibroids, particularly for fertility preservation. It reduces risks such as uterine perforation, thermal damage, and intrauterine adhesions, while allowing for complete resection in a single session. This technique preserves myometrial integrity, improves surgical outcomes, and reduces the psychological and economic burden on patients. While further studies are needed to assess long-term fertility and obstetric outcomes, the cold loop method represents a significant advancement in gynecological surgery.

Healthcare professionals must also be mindful of medico-legal considerations, ensuring clear communication and informed consent to mitigate risks and protect both patients and practitioners.

## 6. Limitations

This study naturally presents some limitations. Since it was a narrative review rather than a systematic one, this could have introduced selection bias. Furthermore, we observed that the included studies showed a high degree of heterogeneity in terms of the sample size, patient characteristics, surgical techniques, and outcome parameters, which limited the generalizability of the conclusions. It was also important to consider the potential publication bias common to all review studies, as studies with positive results are more likely to be published and indexed in the databases used, while those with negative results are less likely to be published. Additionally, the search was limited to publications between 2011 and 2024; this may have excluded earlier relevant work or unpublished data, although we considered that studies published before 2011 might involve less precise surgical evaluations. In conclusion, while the paper does have certain limitations, these can be addressed in our future research.

## Figures and Tables

**Table 1:** STEPW Classification.

	SIZE	TOPOGRAPHY	EXTENSION OF THE BASE	PENETRATION	LATERAL WALL
**0**	<2	Low	<1/3	0	+1
**1**	2 to 5	Middle	1/3 to 2/3	<50%	+1
**2**	>5	Upper	>2/3	>50%	+1

## Data Availability

The raw data supporting the conclusions of this article will be made available by the authors on request.
